# A deep learning based cognitive model to probe the relation between psychophysics and electrophysiology of flicker stimulus

**DOI:** 10.1186/s40708-024-00231-0

**Published:** 2024-07-10

**Authors:** Keerthi S. Chandran, Kuntal Ghosh

**Affiliations:** 1 Center for Soft Computing Research, Indian Statistical Institue, 203 BT Road, Kolkata, West Bengal 700108 India; 2 Machine Intelligence Unit, Indian Statistical Institute, 203 BT Road, Kolkata, West Bengal 700108 India

**Keywords:** Flicker stimulus, Flicker fusion, Electrophysiology, Psychophysics, Deep learning

## Abstract

The flicker stimulus is a visual stimulus of intermittent illumination. A flicker stimulus can appear flickering or steady to a human subject, depending on the physical parameters associated with the stimulus. When the flickering light appears steady, flicker fusion is said to have occurred. This work aims to bridge the gap between the psychophysics of flicker fusion and the electrophysiology associated with flicker stimulus through a Deep Learning based computational model of flicker perception. Convolutional Recurrent Neural Networks (CRNNs) were trained with psychophysics data of flicker stimulus obtained from a human subject. We claim that many of the reported features of electrophysiology of the flicker stimulus, including the presence of fundamentals and harmonics of the stimulus, can be explained as the result of a temporal convolution operation on the flicker stimulus. We further show that the convolution layer output of a CRNN trained with psychophysics data is more responsive to specific frequencies as in human EEG response to flicker, and the convolution layer of a trained CRNN can give a nearly sinusoidal output for 10 hertz flicker stimulus as reported for some human subjects.

## Introduction

Human visual psychophysics is a field of research that employs specialized methods, generating several established findings [1]. The flicker stimulus, which is a stimulus with intermittent illumination, is one such stimulus used in visual psychophysics. Under a given circumstance, a flicker stimulus may appear steady or flickering to a human subject depending on a number of parameters like size, shape, luminance, color composition, and the temporal waveform of the stimulus [2]. When a flickering stimulus no longer appears flickering but appears steady, the flicker is said to be fused, or flicker fusion has occurred. The analysis of perceptual processes by studying the effect on a subject’s experience or behavior by systematically varying the properties of a stimulus along one or more physical dimensions is termed psychophysics [3]. The psychophysics of flicker perception has been studied for the last two and a half centuries [4]. A related domain in the study of flicker perception is the electrophysiological signals of the cortical activities induced by the flicker stimulus. The electrophysiological signals can be the scalp EEG of humans or invasive recordings from the cortex of animals like cats [5, 6]. Although pyschophysics and EEG use completely different methodologies to explain the processing of signals within the brain, there have not been many attempts to integrate, combine, or compliment these two behavioral (macro) and electrophysiological (micro) viewpoints in order to better understand brain signal processing. So far, there does not seem to be much of a relationship between the electrophysiology and psychophysics of flicker stimuli [7]. The present work attempts to bridge this gap between the two domains through a Deep Neural Network (DNN) based computational model of flicker fusion that provides an explanation for some important features of the electrophysiological response to flicker stimuli. We have shown that the intermediate layers of the DNN may show features of the electrophysiological response to the stimulus.

### Psychophysics of flicker perception

**Fig. 1:**
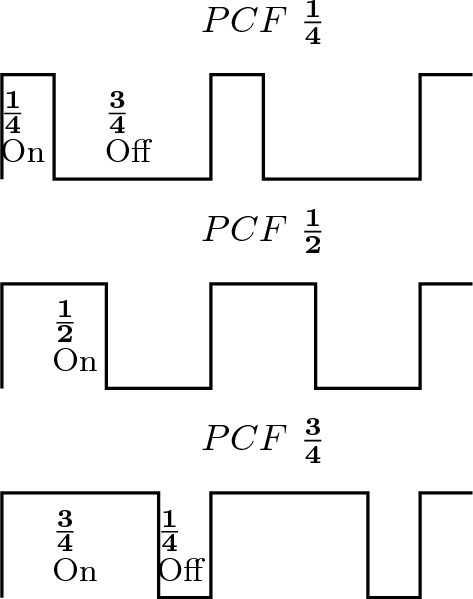
A diagrammatic representation of three photic pulses as variations of intensity with time. The photic pulses have the same frequency or time periods but three different PCFs

The flicker stimuli commonly used in psychophysics experiments on flicker perception are those of sinusoidal and rectangular waveforms [4]. For a sinusodial flicker, the intensity *I*(*t*) at time *t* is given by equation $I(t)=I(1+{\textbf {m}}sin({\omega }t))$, where $0<{\textbf {m}}<1$ and $\omega ={2\pi }{f}$ where *f* is the frequency of the stimulus [4]. The flicker stimulus of rectangular waveforms consists of photic pulses occurring in regular on-off patterns. A flicker stimulus of a rectangular waveform can be expressed by three parameters, which are its intensity or luminance, its frequency, and Pulse to Cycle Fraction(PCF) [8]. Pulse to Cycle Fraction is the ratio of time duration of a pulse to that of the total cycle. PCF is same duty cycle of a rectangular signal [8]. A rectangular waveform with PCF $\frac{1}{2}$ will be a square wave stimulus. A diagrammatic representation of PCF is in Fig. 1. An important numerical parameter in the psychophysics of flicker fusion is the Critical Flicker Frequency(**CFF**). Critical flicker frequency is the number of photic pulses per second needed to eliminate the sensation of flicker [8].

### Electrophysiology of flicker perception

The electrophysiological response to flicker stimuli at areas 17 and 18 of the cat cortex has been recorded as Multi Unit Activities(MUAs) and Local Field Potentials (LFP) by Rager and Singer [6]. The anesthesized cats retinas were exposed to flicker stimuli in screen in front of them [6]. Both LFPs and MUAs thus recorded for a flicker stimulus of a particular frequency shows the fundamental amplitude associated with the flicker stimulus as well as its harmonics[6]. In contrast to multiunit activities components of lower frequencies were present in the LFPs though there is no evidence that they could be subharmonics [6]. Herrmann did a study with ten subjects in which the subjects were exposed to flicker stimulus using specially designed goggles, and their EEG responses to flicker were measured. The diagrammatic representation of the light pulse by Herrmann showed square waves. The human EEG response to flicker stimulus with ten subjects shows the fundamental, hamonics as well as the first subharmonic of the flicker stimulus [5]. The EEG response also shows resonances for stimuli around 10, 20, 40 and 80 hertz [5]. The oscillations are evoked up to 90 hertz and are evoked even when there is no conscious perception of flicker [5]. The average of the fundamental frequency of the evoked EEG response in ten subjects showed a clear resonance for 10 hertz flicker stimulus [5]. van der Tweel had detected a clear sinusoidal response near 10 hertz stimulus in some subjects [7]. In another study with ten subjects that used both square and sinusoidal flicker, the subharmonic frequencies were hardly detectable in occipital electrodes but were instead detected in parietal electrodes [9]. In the same study, subharmonics were detected only in eight of the ten subjects [9].

### Prevailing explanations for EEG response to flicker stimulus

There are two hypotheses on the origin of Steady State Visually Evoked Potentials (SSVEPs) to flicker stimulus: the entrainment of brain oscillators and the superposition of Event Related Potentials(ERPS) [10–12]. It has been assumed by many authors that the harmonics and subharmonics in electrophysiological responses to flicker are generated by nonlinearities in the visual system [9]. The source of these nonlinearities has never been demonstrated conclusively [9]. Previous modeling of EEG response to human stimulus involved feeding periodic signals to models of cortex like the corticothlamic model and the neural mass model [9, 13–15]. Labecki et al. have shown that harmonics and subharmonic responses can be generated by feeding the representation of flicker stimulus to a neural mass model with inhibitory and excitatory neurons [9].

### Deep learning

Machine learning denotes an algorithm that is able to learn from data [16]. Deep learning is a subset of machine learning [16] and Artificial Neural Networks(ANN) that has developed in recent times. While ANN and deep learning models are engineering systems inspired by the biological brain, they are not designed to be realistic models of biological function [16].

### Aim of the current paper

This work aims to connect the relation between pychophysics and electrophysiology of a human subject through a Deep Neural Network (DNN) based computational model that will be trained with pscychophysics data of flicker fusion of a human subject, and no characteristics of EEG data obtained from human subjects will be taken into consideration while training the network. While the network will be trained by backpropagation, it does not imply that any equivalent computational mechanism in the human visual system was similarly trained by backpropagation. The biological brains are instead the product of evolution by natural selection. The flicker stimuli with controlled waveforms used in laboratories are not something that occurs naturally but are instead artificial stimuli used to understand the human visual system which is a product of biological evolution.

### The methodology of the work

The first work to model the computational activity in the brain using Artificial Neural Networks (ANN) via backpropagation was by Zipser and Andersen [17], who modeled the neuronal activity in the parietal cortex of monkey brain, used for calculating the head centered coordinates of the external objects from the position of their images in the retina and slope of the eyes [17]. The outputs of the internal hidden layers of an ANN trained to mimic the same task via supervised learning, gave outputs which were visually similar to the electrophysiological readings obtained from parietal neurons of the monkeys performing the same task. This was not the case with untrained ANNs with random weights [17]. The position of an external object in the world for the purpose of calculating its head-centered coordinates is static, and the task is a problem in static vision. But in experiments of flicker fusion the stimulus is kept fixed at a particular location, and the stimulus intensity varies with time. In a flicker fusion experiment, where the size, shape, and position of the stimulus are invariant with time and the only variant parameter is the intensity, the stimulus can be represented as a timeseries of intensities. Since a human subject classifies the stimulus as flickering or steady, the output can be represented as a binary classification. The input representation will be in the form of a sequence of intensities sampled over time. Recurrent neural networks, which are a family of neural networks for processing sequential data [16], will be used to model flicker phenomena. The human visual system performs low pass and band operations on flicker stimulus [18]. The mammalian retina can also generate transient on and off responses to change in luminance [19]. Receptive field based convolution filters can also detect sudden changes in intensity as is their use for edge detection [20]. Low pass and band pass operations can be mathematically achieved using a linear filter through convolution sum [21]. This work will train Convolutional Recurrent Neural Networks (CRNNs), which consist of convolution layer followed by a recurrent and dense layer [22]. The output of convolution layer of the trained CRNN could be subjectively compared to the features of the EEG response to flicker stimulus.

### Contributions

- The work shows that the fundamental and harmonics of a flicker stimulus can be elicited by a temporal convolution operation on the stimulus.
- The convolution layer of a CRNN trained on psychophsyics data will be more sensitive to particular frequencies, similar to the human EEG response to flicker.
- A pure sinusoidal output can be elicited for a 10 hertz flicker stimulus from the convolution layer of CRNN trained on psychophysics data.

## Related work

### Entrainment

Entrainment is a phenomenon where two oscillators interact with each other to synchronize their oscillations [23]. These synchronizations can enhance or negate each other’s effects as well as synergize with each other to achieve amplitudes greater than the sum of amplitudes of both the oscillators [23]. The frequency of one or both the oscillating systems can be altered so that they become phase locked, or the phase difference between the oscillating systems remains invariant in time, and robust to perturbations [23]. The interaction between the oscillators can be linear or non-linear, and oscillations are merely superimposed with each other. Kirschfeld found that phase of alpha wave oscillations is not affected by flash evoked potentials, and the flash evoked potentials are superimposed on alpha waves without resetting their phase [24]. Schwab et al. found evidence of entrainment to flicker stimulus in both EEG and magnetoencephelogram (MEG) with stronger frequency entrainment in MEG when compared to EEG [12]. Notbohm et al. found evidence of entrainment in EEG response to flicker stimulus but not to the superposition of event related potentials [11]. Gulbinaite et al. showed that flicker stimulus with frequencies near the alpha oscillation can impair stimulus processing in a selective attention task [25]. MEG study by Duecker et al. found that gamma oscillations in the human brain and EEG response to flicker stimulus are evoked in different areas of the human visual cortex [26].

### Role of attention

Flicker stimulus elicits human EEG response in two separate cortical networks depending on attention and temporal frequency [27]. The effect of attention on resonance to flicker stimulus is negative for flicker stimulus with frequencies in the alpha band and positive for stimulus with frequencies in the gamma band [28].

### Deep learning as modeling tool for biological vision

Deep Neural Networks have been put forward as a tool for modeling the brain, in addition to their ability to accurately solve engineering problems through a data-driven approach [29]. Further, deep neural network models of cognition are able to make falsifiable predictions [30]. The outputs from mid layers of deep neural networks, trained for object recognition tasks, are able to model spiking activities in area V1 of monkeys, and Deep Neural Network (DNN) models have been able to predict spiking activity in V1 area of monkeys better than the previous models [31]. Earlier studies have indicated correlations between the output of hidden layers of a trained Alexnet and brain electrode readings from monkeys presented with the same stimulus [32]. The outputs of intermediate layers of a DNN trained on object classification, gives responses similar to neural responses of inferior temporal cortex and area V4 of rhesus macaques presented with the same task. This occurs in spite of the fact that the DNN was not constrained with neural data [33]. The fMRI data of human subjects who viewed natural images has shown that the early visual areas are best explained by shallow models whereas the ventral stream (for object recognition) is best explained by the higher layers of a deep convolutional network [34].

## Materials and methods

### Materials

The psychophysics data previously published in [35], but never used in training any neural network or any other brain computational model to the best of our knowledge, was used in training our proposed model. In this work [35], the CFF was established by the method of limits. The published data for an observer S. H. seen in Table 1, was used for training the neural networks. The flickers in [35] were produced on an opal glass surface, and the subject classified the stimulus as either flickering or a steady after observing it for ten seconds. The stimulus target subtended a visual angle of 2$^{\circ }$ 5’ with the vertical and 4$^{\circ }$ 5’ with the horizontal. The CFFs were obtained for PCFs $\frac{1}{6}$, $\frac{2}{3}$, $\frac{1}{2}$, $\frac{2}{3}$, $\frac{5}{6}$. The CFFs for five different PCFs were obtained for intensities 5340 $cd/ft^2$, 534 $cd/ft^2$, 53.4 $cd/ft^2$, 5.34 $cd/ft^2$ and 0.53 $cd/ft^2$ [35]. The CFFs can be seen in Table 1.

**Table 1:** The CFFs for photic pulses for five different intensities and five PCFs for an observer S.H. (in *hertz*) pulished by Nelson et al. [35]

Intensity $(cd/ft^2)$	PCF $\frac{1}{6}$	PCF $\frac{1}{3}$	PCF $\frac{1}{2}$	PCF $\frac{2}{3}$	PCF $\frac{5}{6}$
5340	58.17	51.83	59.17	52.5	47.00
534	48.0	50.0	50.83	48.7	46.17
53.4	34.67	36.17	36.33	35.2	32.67
5.34	23.17	27.	25.67	25.2	20.83
0.53	17.17	19.0	17.83	19.0	15.67

### Methods

#### Model

A photic pulse can be represented as a timeseries array of intensities. The retina can have on pathways, off pathways as well as transient on and transient off cells [19]. The on pathways are activated when there is light falling on them, and off pathways are activated when there is no light falling on them. In addition to these, there are transient on and off pathways which are activated when there is a sudden change in the intensity of light falling on them. The transient on and off operations can be mathematically represented by linear filtering operations. The machine could thus mimic the transient on and off pathways by performing a convolution operation on the signal. While the machine stores the whole representation of the photic pulse in memory while doing the convolution operation, that will not be the case in reality. Only the intensities in the immediate past will be necessary for the human visual system to perform transient on and off operations. The output of the convolution layer is fed into a recurrent layer. Recurrent neural networks are a family of neural networks for processing sequential data [16]. The final state of the recurrent layer is fed into a Multi Layer Perceptron for classification.

Python based Keras library was used to train and run the model. Loss function sparse_categorical_crossentropy with Adam optimizer was used to train the model. Cross entropy is a loss function which is used in classification problems. The cross entropy H for a datapoint is given by equation $H(p,q)=-\sum _{x{\in }classes}^{}p(x)log(q(x))$ where *p*(*x*) is the true probability distribution of the classes and *q*(*x*) is the probability distrbution of the classes predicted by the model [36]. Sparse categorical cross entropy was used to provide labels as integers. Time series representation of flicker stimulus with desired PCF and intensity can be generated as one dimensional arrays. These could be assigned the labels flickering or fused based on psychophysics data.

#### Minibatch generation

**Fig. 2:**
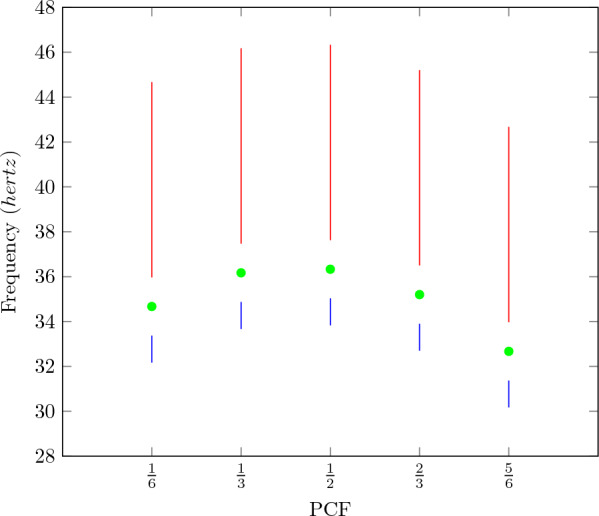
The frequency ranges used to select the flickering and fused datapoints for photic pulses with intensity 53.4 $cd/ft^2$, for training the network. The photic pulses with a particular frequency and PCF lying in the blue line were assigned the label ’flickering’. Those in the red line were assigned the label ’fused’. The corresponding CFFs for the five PCFs have been marked in green

The time series data with a desired PCF and frequency were generated with the Python library function *scipy*.*signal*.*square*(). The square waves so generated will have two values -1 and 1 in them. By adding the value 1 to that wave, and then multiplying by 0.5, followed by further multiplication by the intensity value of the wave we get the sampled representation of a wave with the desired intensity.

**Table 2:** Frequency ranges or constant amplitudes associated with the photic pulses in a minibatch for training and validation for a particular label and PCF

	Label Fused	Label Flickering
Validation data (n=50)	n waves with frequency between CFF+1.3 to 100 Hz	n waves with frequency between CFF$-2.5$ to CFF$-1.3$ Hz
Training data (n=10)	50% probability of waves with frequency CFF+1.3 to CFF+10 Hz	n waves with frequency between CFF$-2.5$ to CFF-1.3Hz
	25% probability of a continuous signal with with value between intensity of wave and zero	
	25% probability of a continuous signal with value 0	

We have interpolated the data based on the assumption that for a frequency above CFF for a particular PCF, the photic pulse will appear as fused to the subject, and for frequencies below that CFF, it will appear as flickering. Waves with frequencies just below CFF were assigned the label of flickering. Training data and validation data were generated from scratch in each iteration during the training process. Waves with frequencies between CFF$-1.3$ and CFF$-2.5$ were assigned the label flickering for each PCF. The frequency interval in which photic pulses were assigned the label flickering was kept small, as a photic pulse of long duration is indistinguishable from a steady source of light. We had no way to estimate that duration. Waves with frequencies between CFF+1.3 and CFF+10 were assigned the label non-flickering. This was done as any stimulus above the CFF will be perceived as non-flickering or fused. A constant non-flickering stimulus is indistinguishable from a fused flicker stimulus. So constant stimuli with amplitude between intensity of the flicker stimulus and 0 $cd/ft^2$, as well as a complete dark stimuli with 0 $cd/ft^2$ throughout it, were used in training dataset with the label fused. A diagrammatic representation of data ranges used for training for intensity 53.4 $cd/ft^2$ is seen in Fig. 2.

For validation, waves with frequencies of CFF+1.3 to CFF+100 were assigned the label fused. This was because photic pulses with frequencies above the CFF should be labeled as fused or steady by a subject. Waves with frequencies between CFF$-1.3$ and CFF$-2.5$ were used with label flickering for validation as well. The trained neural network should detect a time series of constant amplitudes as fused. Since we did not know the minimum time period for which the neural network will classify a constant amplitude series as fused, the frequency range of photic pulses used in validating the neural network was kept the same as that was used for training the neural network. For a training minibatch, ten photic pulses each  for both flicker and fused, were generated for all PCFs. For five PCFs and two labels, it provided 100 samples. Similarly, 50 photic pulses with label flicker and fused were generated for each PCF in validation minibatch. It provided 500 photic pulses for validation minibatch in each iteration. New minibatches were generated for each iteration by randomly sampling from the frequency range in both Experiment 1 and Experiment2 described in the next sections. The training and validation data for each iteration is shown in Table 2. In Table 2,*n* is the number of photic pulses generated for a class with a particular PCF in a minibatch. The total number of samples in the minibatch will be 10 times *n,* as there are 5 PCFs and two classes.

## Experiments and results

### Experiment 1

Only the photic pulses with the middlemost intensity of 53.4 $cd/ft^2$ were used for training and testing in this experiment. Photic pulses of desired frequency and PCF with intensity 53.4 $cd/ft^2$ were generated with the method mentioned before for 12 seconds duration and sampled at 1 milliseconds. From this array, a smaller array of 7168 elements was selected from one of the first 3000 elements chosen at random. This was done to make the last element in the photic pulse irrelevant during the training process. The classification of a photic pulse is independent of the fact whether the last element is of intensity 0 $cd/ft^2$ or some other intensity. From this representation of a photic pulse with 7168 elements, one of the first 500 elements was chosen at random. The elements in the array from index 0 to that of the chosen element were filled with random intensity between 0 and the intensity of the photic pulse, which is 53.4 $cd/ft^2$. This was done under the assumption that for a photic pulse of a finite enough time duration, some small perturbations at the start of the photic pulse will not have any effect on the final classification after a prolonged time period.

The neural network model used in the experiment is shown in Table 3. Eight convolution filters of 120 elements were used in layer 1. Biases were used in each layer. Activation function Leaky ReLU with default parameters (alpha=0.3) from the TensorFlow library was used for convolution operation (layer 1). Valid convolution was performed in the convolution layer.

**Table 3:** Structure of neural network used in Experiment 1

Layer	Dimension	Dropout	Activation function	Number of paramenters
Layer 0 (Input Layer)	[None, 7168, 1]			
Layer 1 (1 dimensional convolution)	[None, 7049, 8 ]		leaky ReLU	968
Layer 2 (RNN Basic Cell Final State)	[None, 8]	0.5	sigmoid	136
Layer 3 (Dense Layer) (Dense Layer)	[None, 8]	0.5	sigmoid	72
Layer 4 (output Layer) (output Layer)	[None, 2]		softmax	18

### Results of experiment 1

For testing, photic pulses of desired frequency, intensity 53.4 $cd/ft^2$ and PCF $\frac{1}{2}$ generated for a duration of 7.168 seconds, sampled at a frequency of 1 millisecond, were generated by scipy.signal.square function from the Python library. They were fed to the input layer (Layer 0). For a neural network with random weights, no noticeable differences could be observed for convolution outputs for 10 hertz or 20 hertz stimuli and stimuli of other frequencies. The set of weights for iteration with the lowest loss was chosen. The weights after iteration 1582, with a loss of 0.1153 and an accuracy of 0.972 for validation dataset were loaded into the network.

#### Output for 10 hertz signal

**Fig. 3:**
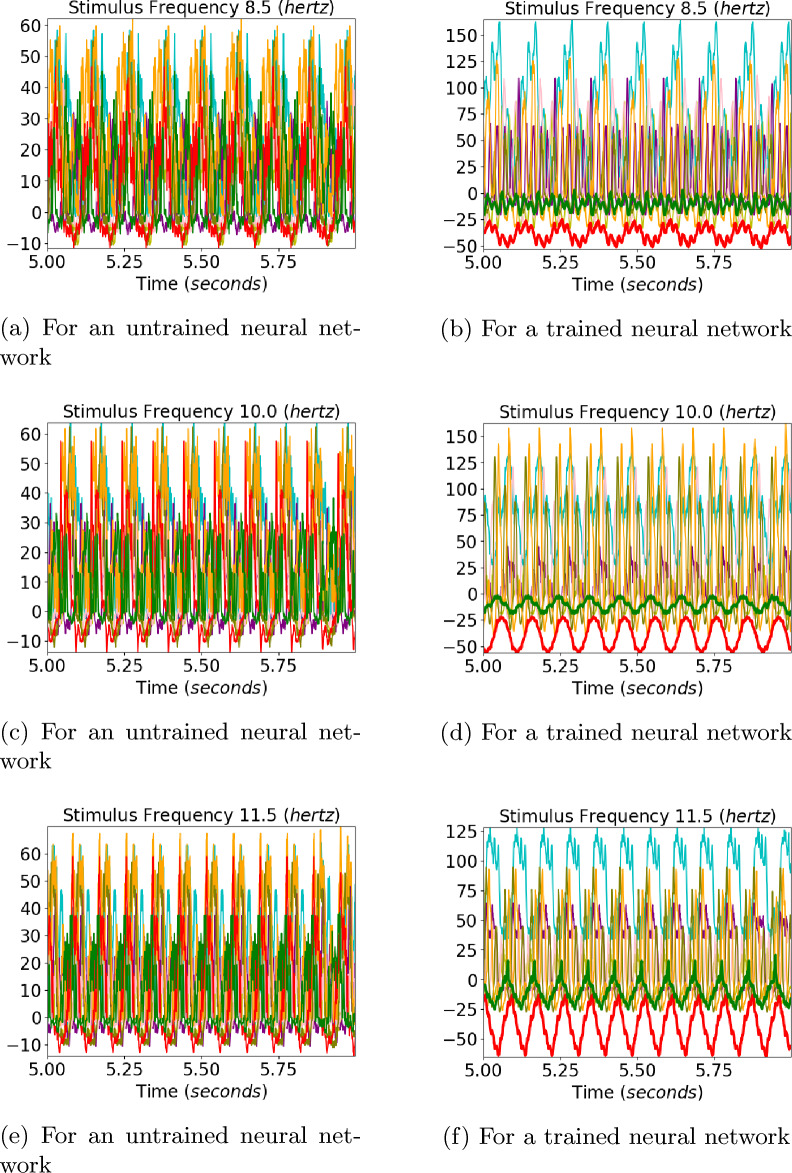
Convolution layer outputs of the eight different neurons for input stimulus representations for 8.5, 10, and 11.5 hertz square waves are shown in the figure. The left side images show the output of an untrained neural network and the right side images show the output for the trained neural network. Sinusoidal outputs can be seen in the output to 10 hertz square wave inputs for the trained network for neurons 3 and 6, marked by red and green colors

The output of a convolution layer for an untrained neural network can be seen in Figs. 3a, 3c, and 3e for input stimuli of frequencies 8.5, 10, and 11.5 *hertz*. No particular resonance can be seen for a stimulus of frequency 10 hertz.

**Fig. 4:**
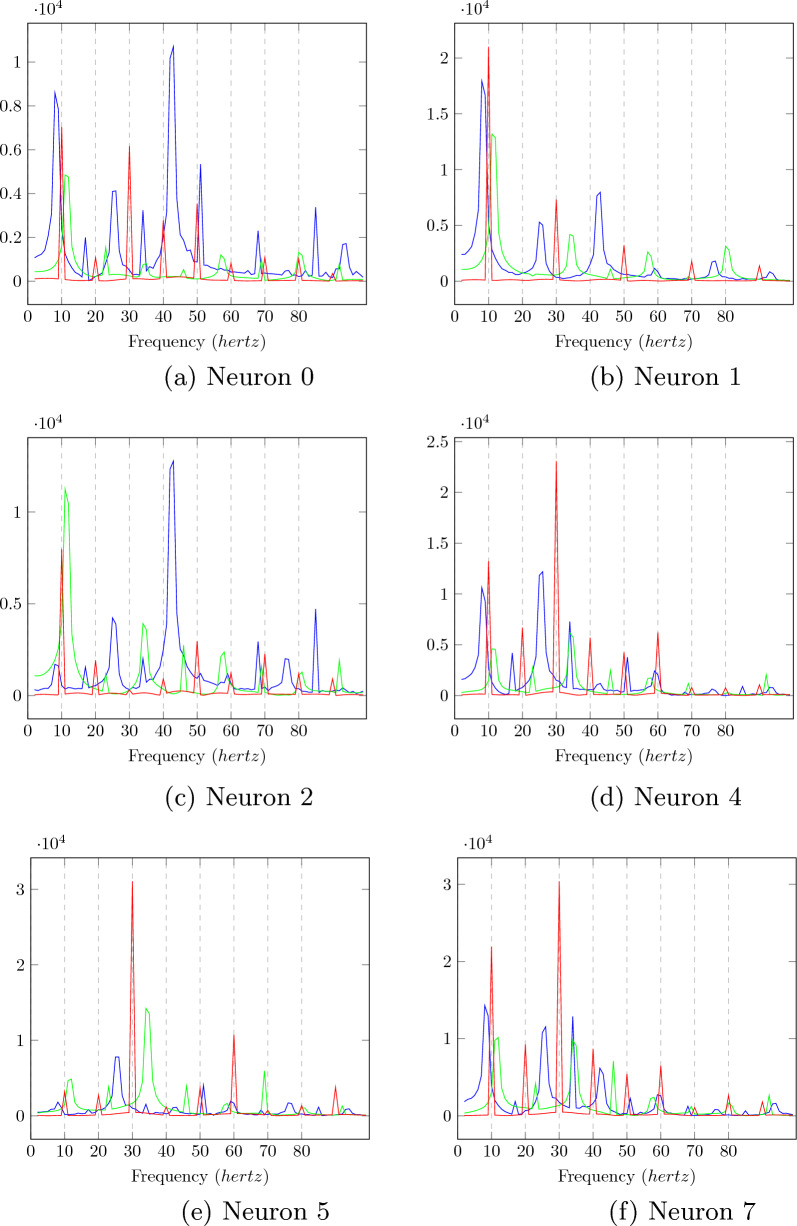
The amplitudes of the Fourier transform in the convolution output of the trained neural network for the six neurons whose convolution outputs did not show clear sinusoidal patterns for the 10 hertz signal. The Fourier amplitudes for 8.5, 10, and 11.5 hertz have been plotted in blue, red, and green, respectively. We can see that multiple peaks can be observed for the 10 hertz signal at subharmonics of 10 hertz

**Fig. 5:**
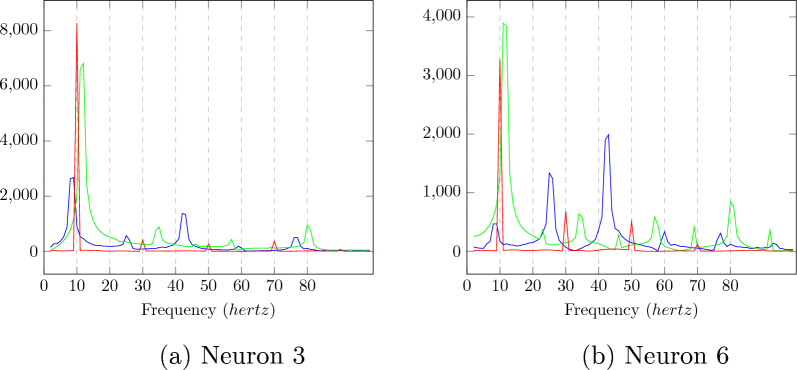
The amplitudes of the Fourier transform of the convolution output from the trained neural network for the six neurons whose convolution outputs did showed sinusoidal patterns for the ten hertz signal. The fourier amplitudes for 8.5,10, and 11 hertz have been plotted in blue, red, and green. The peaks at subharmonics for 10 hertz are much lower than the fundamental frequency for the output of a ten hertz signal

**Fig. 6:**
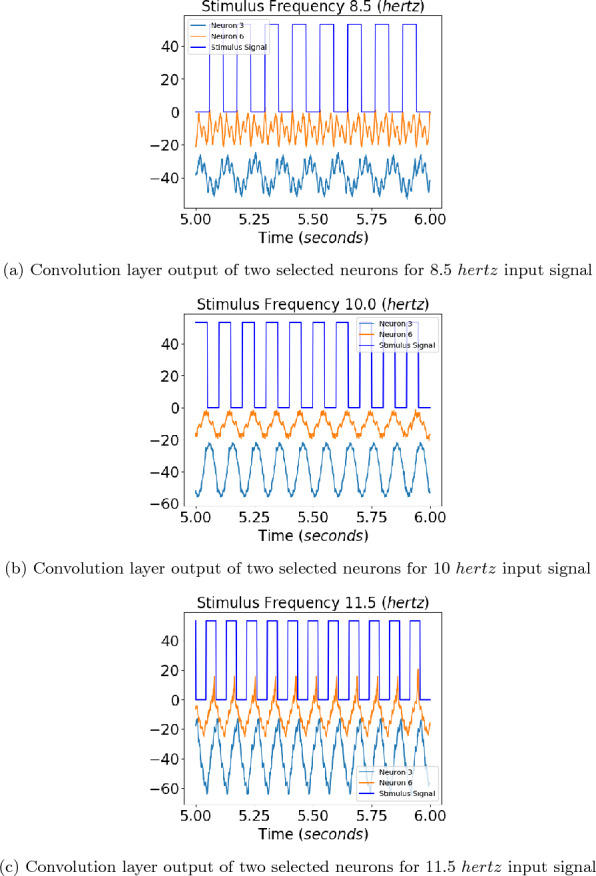
The convolution layer output of the trained neural network used in Experiment 1 for two selected neurons for 8.5, 10, and 11.5 hertz signals. A clear sinusoidal output could be observed for the 10 hertz signal which is not the case with 8.5 or 11.5 hertz signals

The outputs of a convolution layer for a trained neural network can be seen in Fig. 3b, d, and f for input stimuli of frequencies 8.5, 10, and 11.5 *hertz*. Sinusoidal ouputs can be observed for 10 *hertz* input in Fig. 3d which cannot be seen for 8.5 *hertz* input and 11.5 *hertz* input in Fig. 3b and f. The outputs of neurons that gave sinusoidal outputs at ten hertz have been plotted with red and green lines. For a pure sinusoidal signal, its fourier transform will have amplitudes at the frequency of the signal alone. It can be seen that for a ten hertz signal, the six neurons whose Fourier amplitudes have been plotted in Fig. 4 are not sinusoidal. For the two neurons that showed sinusoidal output at ten hertz stimulus, the neurons showed a prominent peak at 10 hertz in fourier transform compared to subharmonics, as can be seen in Fig. 5. The The output of two neurons for which the sinusoidal response at 10 hertz is prominent can be observed from Fig. 6a, b, c.

#### Profile of fundamental frequency

**Fig. 7:**
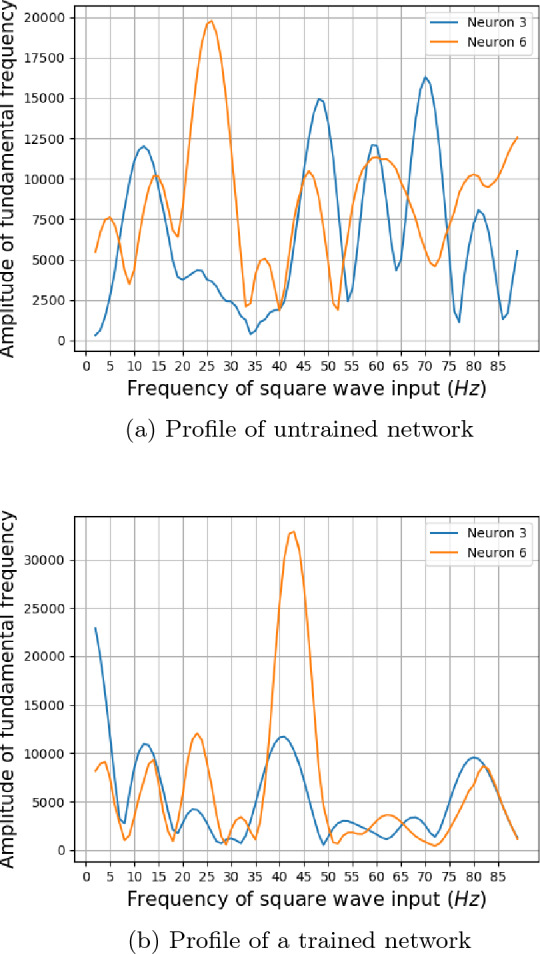
Profile of the fundamental for the two neurons that gave sinusoidal output for 10 hertz stimulus. Previous studies have shown that the human EEG output of individual subjects gave three distinct peaks around 10 hertz and in 20–30 hertz and 40–50 hertz ranges [5]. The subjective comparisons show that the profile of fundamental frequency for a trained network is closer to that of human EEG when compared to that of an untrained network, which is having a lesser number of peaks

**Fig. 8:**
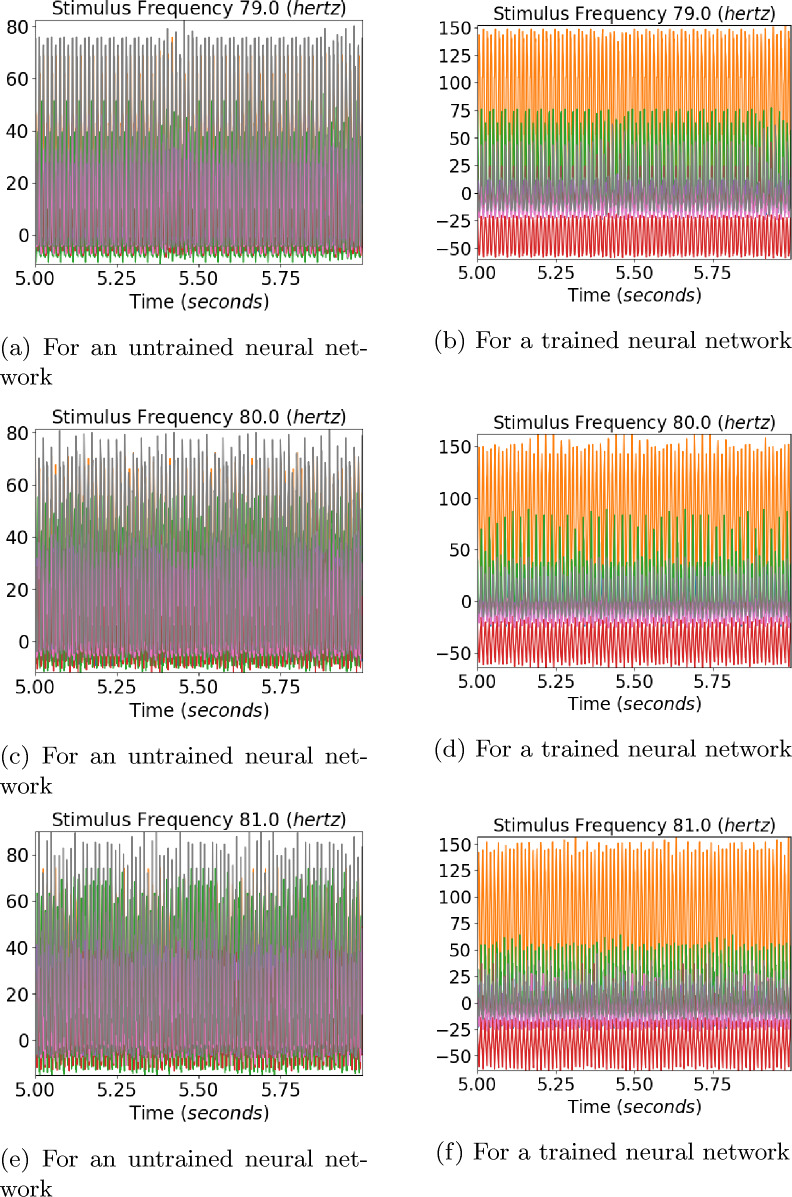
Convolution layer outputs for input stimulus representations for 79, 80, and 81 hertz. Previous studies have found a 10 hertz component in human EEG reponse to 80 hertz flicker [5]. Two envelopes with a low frequency can be seen in output of the trained network for 80 hertz stimulus

The amplitude associated with the fundamental frequency of a subject as plotted by Herrmann showed clear resonance peaks around 10, 20, and 40 hertz [5]. The amplitude of fundamentals associated with neurons 3 and 6, which showed sinusoidal responses for ten hertz, can be seen in Fig. 7. The profile of the trained network shown in Fig. 7b shows four distinct peaks around 10, 20, 40, and 80 hertz for the neurons. This is in contrast with the profile for an untrained network in Fig. 7a, which shows numerous peaks but no distinct resonance peaks. Previous works on human EEG response to flicker stimulus have shown that the average of the fundamental frequency across 10 subjects exhibited strong resonance peaks around 10 hertz and weaker peaks in the 20–30 hertz and 40-50 hertz ranges [5]. The data for a single subject consisted of only one peak in the mentioned three ranges [5] (Fig. 8).

#### Output for 80 hertz signal

**Fig. 9:**
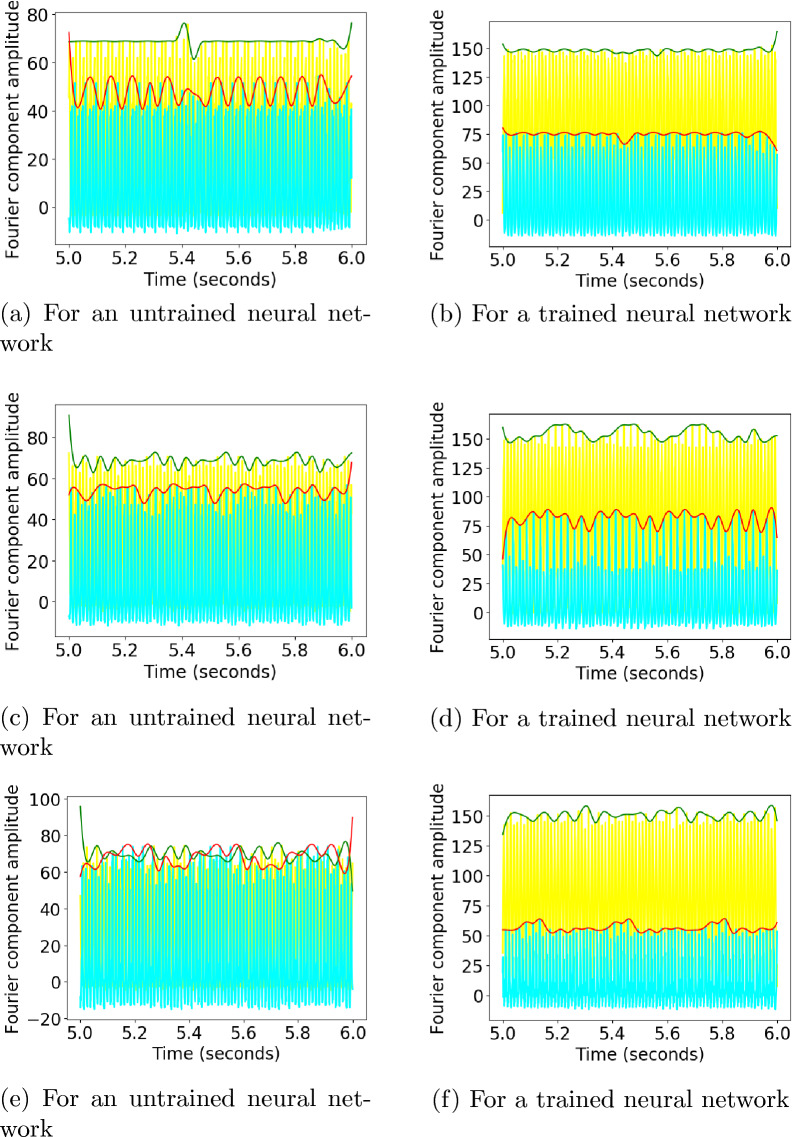
The convolution layer output of the trained neural network used in Experiment 1 for two selected neurons 1 and 2 have been plotted in cyan and yellow for 79, 80 and 81 hertz. An envelope with low frequency can be seen in the convolution output for 80 hertz signal. The envelops have been constructed using maxima sampling and reconstruction of signal using cubic spline interpolation

**Fig. 10:**
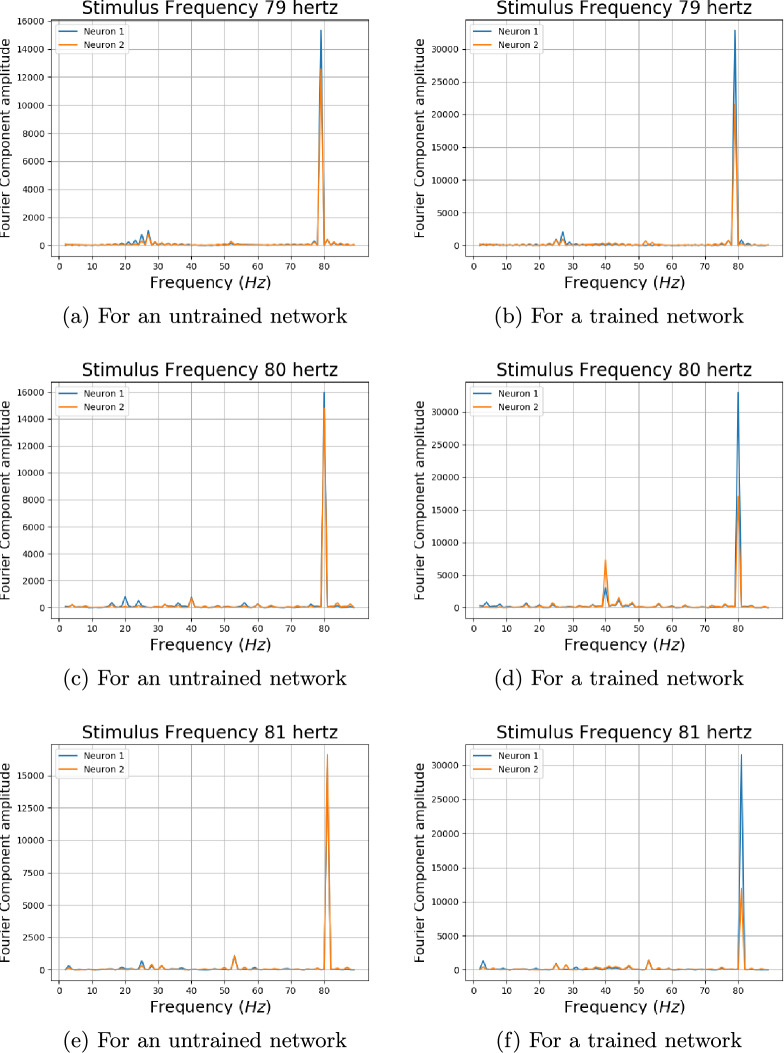
The amplitudes associated with various frequencies in the convolution output for square wave stimulus of frequencies 79, 80 and 81 hertz for both trained and untrained neural network. A clear subharmonic with frequency 40 hertz can be seen in output of 80 hertz stimulus for the trained neural network

An 80 hertz flicker stimulus is known to invoke clear ten hertz response in human EEG which is not present at adjacent frequencies [5]. The output of the convolution layer for an untrained neural network for frequencies 79, 80 and 81 hertz can be seen in Fig. 8a, c, and e. The output of the convolution layer for an trained neural network for frequencies 79, 80 and 81 hertz can be seen in Fig. 8b, d, and f. We could not obtain a sinusoidal 10 hertz response to 80 hertz stimulus as reported in literature. But an envelope which is not observed at 79 and 81 hertz stimulus could be observed for 80 hertz stimulus for some neurons of a trained neural network as can be seen in Fig. 9. The envelopes of a signal can be obtained by extrema sampling followed by signal reconstruction using sampled extrema with methods like Cubic Spline [37]. The envelopes were constructed by the method of sampling the maxima with a sample window of 25 points and signal reconstruction using cubic spline. The amplitudes of the evoked frequencies for stimulus of 79, 80 and 81 hertz for both trained and untrained neural networks can be seen in Fig. 10. A clear subharmonic of 40 hertz can be seen in output of trained network for 80 hertz as can be seen in Fig. 10d while the subharmonic is absent for output of stimulus of 79 and 81 hertz of the same network as can be seen in Fig. 10b and f.

### Experiment 2 and results

The data was trained with a different neural network, having ReLU activation function in the convolution layer. The outputs of convolution layer were added together in a dense layer before going to the recurrent layer. It was done under the assumption that different cells in human visual system can have different temporal responses to a flickering stimulus and they might be present in the same biological layers in the visual system. In human retina the midget and parasol gangellion cells, have different temporal responses [38].

The photic pulse trains were sampled at 0.5 milliseconds to create a representation of waveforms. Square Wave arrays of 22384 elements with desired frequencies were generated. From these an array of length 16384 elements were selected, starting with one of the first 3000 elements chosen at random. The initial elements of this array were chosen at random from a number between 0 and 700. The initial elements of photic pulse representation so generated, was filled with a random intensity between 0 and intensity of the wave. The reason for this randomizations were same as in Experiment 1. These perturbations augment the training data. The whole train represented a photic pulse of duration 8.192 s.

**Table 4:** Structure of neural network used in Experiment 2

Layer	Dimension	Dropout	Kernel constraint	Activation function	Number of paramenters
Layer 0 (Input Layer)	[None, 16384, 1]				
Layer 1 (Convolution Layer)	[None, 16245, 8 ]			ReLU	2248
Layer 2 (Dense Layer)	[None, 16245, 4]		ReLU	ReLU	36
Layer 3 (RNN Basic Cell Final State)	[None, 5]	0.5		Sigmoid	50
Layer 4 (Dense Layer)	[None, 5]	0.5		Sigmoid	30
Layer 5 (Output Layer)	[None, 2]			Softmax	12

The structure of neural network used in the experiment can be seen in Table 4. Bias were used for both dense layers, convolution layer and RNN basic cell. In the dense layer after convolution (layer 2), kernel constraint Rectified Linear Output was applied to the kernel parameters. A convolution layer with a convolution operator length of 280 weights was used in the first layer. It corresponded with a time period of 140 ms.

The loss and accuracy for each iteration was tabulated after training. The losses and accuracies for validation set over iterations were filtered with a gaussian filter of sigma 11. The selected iterations have been listed in Table 5.

**Table 5:** The iteration whose corresponding weights were chosen for neural network

Intensity $cd/ft^2$	Total iterations	Least loss	Selected iteration	Accuracy
5340	4000	0.649	1893	0.6040
534	4000	0.3298	3411	0.89
53.4	4000	0.1065	2128	0.968
5.34	2000	0.0448	1938	0.998
0.53	4000	0.043	3953	0.994

**Fig. 11:**
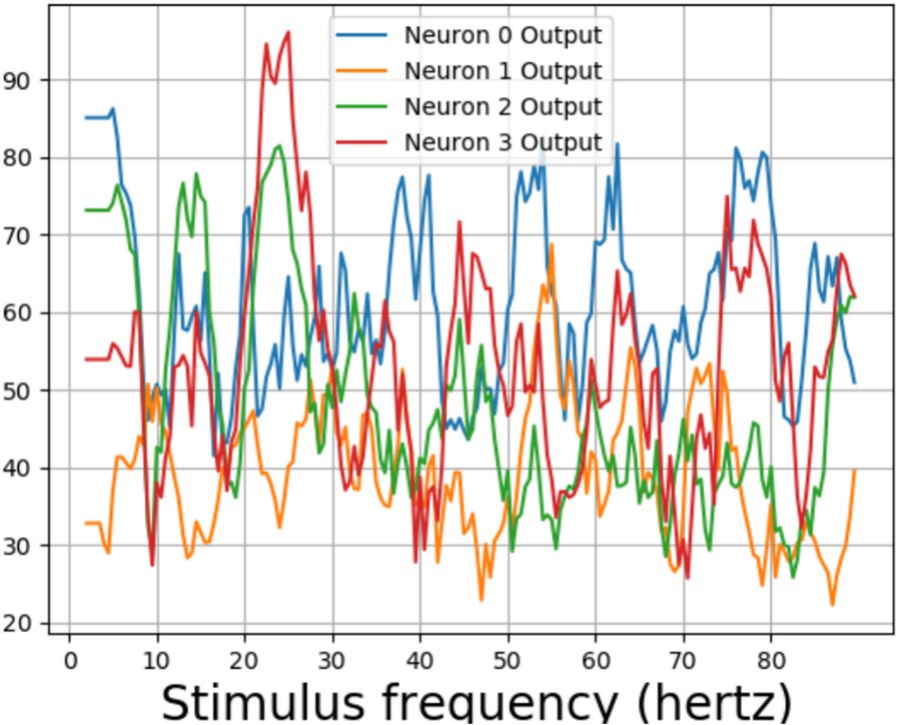
The differences in maximum and minimum amplitudes in a 1 s interval from the output of layer 2 of an untrained neural network used in Experiment 2

**Fig. 12:**
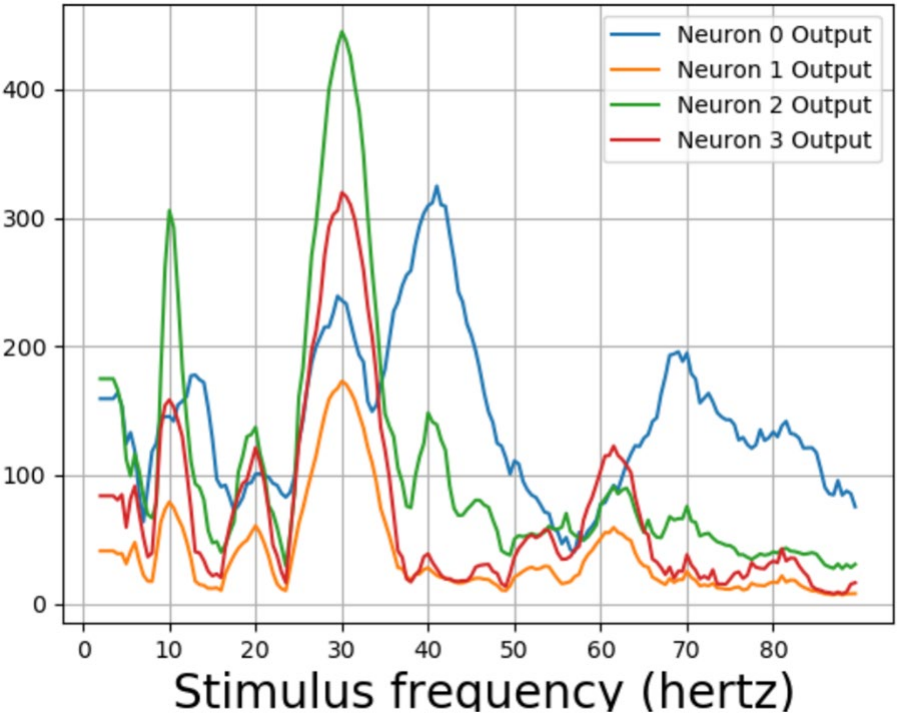
The differences in maximum and minimum amplitudes from the output of layer 2 for 1 s intervals for a neural network trained and tested with 53.4 $cd/ft^2$ photic pulses

**Fig. 13:**
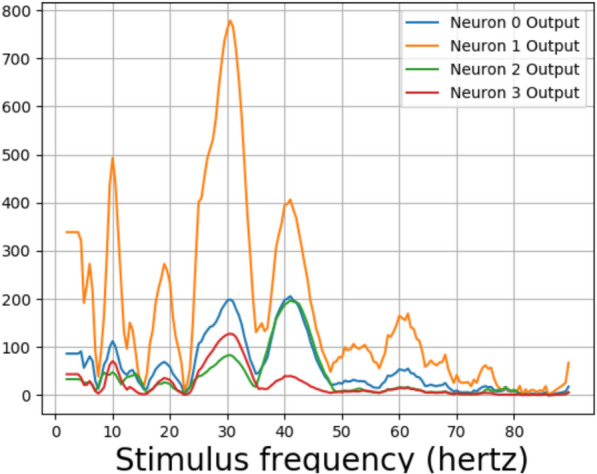
The differences in maximum and minimum amplitudes from the output of layer 2 for 1 s intervals for a neural network trained and tested with 53.4 $cd/ft^2$ photic pulses. The neural network was trained with same method for a second time

**Fig. 14:**
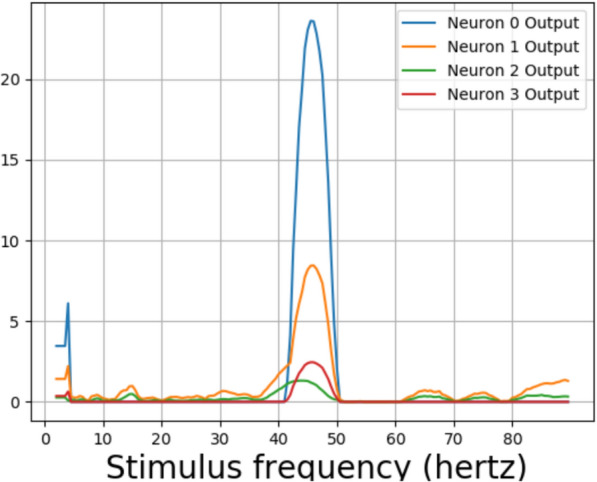
The differences in maximum and minimum amplitudes from the output of layer 2 for 1 s intervals for a neural network trained and tested with 5.34 $cd/ft^2$ photic pulses

For testing, the model waves of intensities 5340 $cd/ft^2$ and 534 $cd/ft^2$ were not considered due to low training accuracies. The difference in maximum and minimum values from layer 2, for one second interval for an untrained neural network for various stimulus frequencies for photic pulses of intensity 53.4 $cd/ft^2$, can be be seen in Fig. 11. The differences from the layer 2 output of the neural network trained again, with 53.4 *hertz* stimuli can be seen in Figs. 12 and 13. A peak can be observed at 10 hertz in both Figures. Peaks could also be seen near 20 hertz and 40 hertz. Some extra peaks including those at 30 hertz were obtained in both the Figures. The output for a neural network trained with 5.34 $cd/ft^2$ for same operation can be seen in Fig. 14. The output did not show peaks at 10, 20, 40 or 80 hertz.

**Fig. 15:**
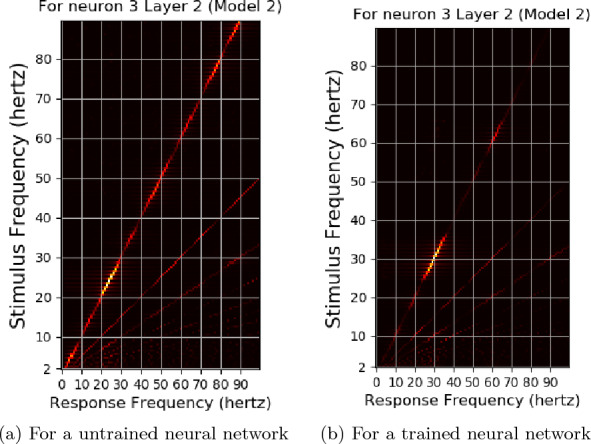
The response frequencies vs stimulus frequencies in output of neural network in Neuron 3 for Layer2

**Fig. 16:**
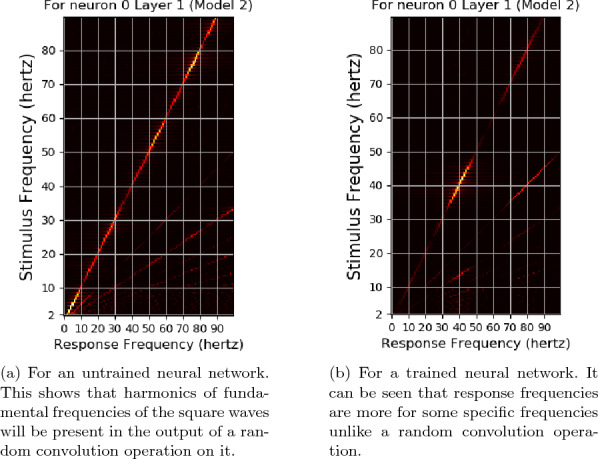
The response frequencies vs stimulus frequencies in output of Neuron 0 of Layer1 of neural network used in Experiment 2

**Fig. 17:**
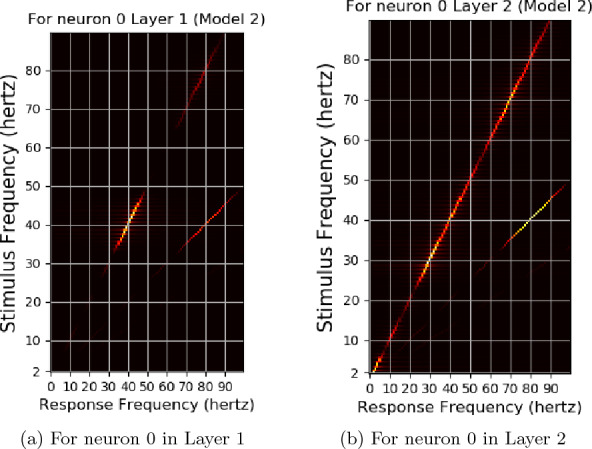
The response frequencies vs stimulus frequencies for a sinusodial stimulus of waveform $I=I_{o}(1+{{\textbf {m}}}sin({\omega }t))$ for the neurons for a neural network of Model 2 trained with photic pulse representations of $54.3cd/ft^2$. Here $I{o}=54.3$, ${\textbf {m}}=0.1$ and ${\omega }=2{\pi }{f}$ where *f* is the the frequency of the stimulus. The subharmonics are present in the output of the sinusoidal stimulus

The stimulus frequencies for square waves that invokes responses for frequencies from 2 to 100 hertz for this neural network for photic pulse of intensity 53.4 $cd/ft^2$ have been plotted in Figs. 15a, b and 16a, b. The present model corresponding to Experiment 2 (refered to as Model 2) predicts fundamental frequencies as well as harmonics in the output of the neural network as can be seen in Figs. 15b and 16b. Even a convolution filter with random weight can generate harmonic frequencies in output of a periodic stimulus as is evident from 16a. It can be seen from the figures that the convolution output of a trained neural network is more sensitive to specific frequencies than an untrained neural network. The output of a trained neural network is similar to human EEG response to flicker in being sensitive to certain frequencies. Further as can be seen in Fig. 17 the fundamental as well as harmonics are evoked even in the sinusoidal response to flicker stimulus.

## Discussion

The findings from the present work indicate that CRNNs can be used to train pyschophysics data of photic pulses for a particular intensity that varies in PCF and frequency. The present work shows that it is possible to obtain a clear sinusoidal response at 10 hertz from a neural network trained with psychophysics data, in spite of the fact that no 10 hertz photic pulse signal or EEG data were used in training the neural network. Clear sinusoidal responses have been previously obtained near 10 hertz for EEG response to flicker [7]. It also shows that the convolution layers of such a CRNN are more likely to show resonant output at some particular frequencies, similar to the human EEG response to flicker. The similarities in output between the convolution layer of the CRNN and the EEG response to the stimulus can be explained by the assumption that a small region in a cortical layer with thousands of neurons in it acts in a manner similar to that of an artificial neuron in an ANN. When neurons present in a cortical layer fire in unison, the electric fields generated by the neurons can be measured outside the brain via EEG. The model put forth in this work that human EEG response to flicker stimulus is a convolution operation on the stimulus is in line with the hypothesis that the response is the superposition of event related potentials as opposed to the entrainment hypothesis.

The main sources of light in the natural world, viz., sun and moon, emit steady light with no sudden change in intensity. The only way the intensity of light emitted from any point in the natural world would change is when there is some motion at that point. The cell assemblies that lead to characteristic response in EEG, to flicker stimuli may have evolved in order to detect motion at particular points.

Flicker fusion can happen at various stages of information processing, beginning with the retina. Here, only one stage of differentiation in the brain was considered. This can be the reason why only one intensity was able to produce an EEG-like response in the intermediate layers of CRNN, trained to imitate the human brain. It could also be a reason why the neural network could not be trained with photic pulses of intensities greater than 53.4 $cd/ft^2$. The intermediate outputs of neural networks trained with intensities lesser than 53.4 $cd/ft^2$ did not show resonances near the reported frequencies where human EEG shows resonant response to flicker stimulus. Also, harmonics and sub-harmonic oscillations, in addition to the fundamental frequency of flicker stimulus have been detected in the human EEG response to flicker stimulus. The sub-harmonic oscillations, unlike harmonic oscillations, were reported to come from the parietal electrodes instead of the occipital electrodes [9]. No clear evidence of sub-harmonic oscillations were reported in local field potentials and multi unit activities of cat visual cortex stimulated with flicker stimulus [6]. The present work has been unable to detect sub-harmonic oscillations in the itenrmediate convolution layers. Digital circuits with flip flops are can be used as frequency doublers to generate a pulse with doubled frequency of the clock pulse [39]. A similar mechanism might be happening in the generation of the sub-harmonics of the fundamental frequencies of the flicker stimulus, with the flicker stimulus acting as a clock signal.

### Limitation and future work

The present work was trained only on the data of a single subject, which is a limitation of this study. But the work does provides support for the methodology that can be tested with psychophysics and EEG data acquired for the purpose in future. The amplitudes of the fundamental and the first and second harmonics in SSVEPs are not stable over time in some human subjects [40]. The present model has been unable to explain this phenomenon. Moreover, we generated training and validation data on the assumption that, for a particular PCF and intensity, the human visual system will perceive all photic pulses with frequencies above the CFF as flickering. However, outliers exist above and below CFF [41]. Also, in the present work, no EEG recordings were made at the time of acquisition of psychophysics data used in training the neural network. A future experiment with both psychophysics data and EEG data collected from the same subject in identical circumstances will possibly be able to ascertain more correlations between the two. The classification for data points represented by intensity, PCF and frequency can be measured for the subject by the method of constant stimulus. A large set of data points for a particular intensity with two parameters, viz. frequency and PCF, labeled into two classes, could be used to train a similar neural network. In spite of some of these limitations mentioned above, the present work provides the first such computational framework that involves training a deep neural network with psychophysics data to predict brain activity at the electrophysiological level.

## Conclusion

The present work used a recurrent neural network based framework to model flicker, a time dependent psychophysics data. We have shown that the intermediate layers of the network could show features of the electrophysiological response to the stimulus. Clear sinusoidal responses could be obtained from intermediate layers of the network for a ten hertz stimulus input although no electrophysiological data was used to train the model. The presence of the fundamental frequency of the flicker stimulus as well as the harmonics can be explained as a temporal convolution operation of the stimulus. We have further shown that the output from convolution layer of a CRNN trained with psychophysics data will be more responsive at particular frequencies, similar to the human EEG response to flicker. The proposed CRNN model could be used to test the relationship between the psychophysics of flicker fusion and the electrophysiology of flicker from a same subject.

## Data Availability

No datasets were generated or analysed during the current study.
